# Comprehensive motion management for liver SBRT on a 1.5 T MR-Linac: Initial clinical experience and dosimetric comparison with an ITV-based workflow

**DOI:** 10.1016/j.ctro.2026.101335

**Published:** 2026-10-24

**Authors:** Monica Lo Russo, Simon Böke, David Mönnich, Moritz Schneider, Elgin Hoffmann, Jessica Boldt, Hathal Haddad, Maximilian Niyazi, Daniela Thorwarth, Cihan Gani, Marcel Nachbar

**Affiliations:** a Department of Radiation Oncology, University Hospital and Medical Faculty, Eberhard Karls University, Tübingen, Germany; b Section for Biomedical Physics, Department of Radiation Oncology, University Hospital and Medical Faculty, Eberhard Karls University Tübingen, Germany; c German Cancer Consortium (DKTK), Partner site Tübingen, and German Cancer Research Center (DKFZ), Heidelberg, Germany

**Keywords:** Liver tumors, MRgRT, Adaptive radiotherapy, Comprehensive Motion Management, CMM

## Abstract

**Background and purpose:**

Respiratory-gated MR-guided workflows enable expiration-gated liver stereotactic body radiotherapy (SBRT) based on real-time intrafraction target monitoring. We evaluated the dosimetric impact and early clinical feasibility of this approach by comparing clinically delivered expiration-gated plans with retrospectively generated non-gated ITV-based plans.

**Methods:**

The first 20 consecutive patients treated with online adaptive MR-guided SBRT for primary or secondary liver malignancies on a 1.5 T MR-Linac between October 2024 and April 2025 were included. Five patients had two lesions, treated with separate plans in two patients, resulting in 22 matched plan pairs. For each clinically delivered gated plan, a retrospective ITV-based plan with identical prescription dose was generated. Paired differences were tested with the Wilcoxon signed-rank test, and *p*-values were adjusted for multiple testing using the Benjamini–Hochberg procedure. The dose–volume analysis was considered exploratory.

**Results:**

Median planning target volume was lower with the gated workflow than with ITV-based planning (28.0 vs. 41.9 cc). Organ-at-risk constraint violations were fewer with the gated workflow (10 vs. 19), with 11 violations resolved and 2 newly occurring. PTV D98% did not differ significantly (*p* = 0.41), although one gated and four ITV-based plans showed PTV D98% < 95%. Uninvolved liver dose was significantly lower with the gated workflow (median Dmean difference 0.97 Gy and 0.93 Gy for three- and five-fraction plans; adjusted *p* ≤ 0.037). Among 93 evaluable fractions, the mean plan-level median active delivery time was 7.6 min for three-fraction and 6.7 min for five-fraction treatments.

**Conclusion:**

Expiration-gated MR-guided liver SBRT reduced target volumes and improved normal tissue sparing without compromising target coverage or routine clinical feasibility.

## Highlights

• Respiratory-gated MR-guided workflow improved uninvolved liver sparing while reducing OAR constraint violations.
• Comparable target coverage was maintained with expiration-gated MR-guided delivery.
• Workflow feasibility was confirmed by acceptable irradiation and treatment times.

## 1 Introduction

Stereotactic body radiotherapy (SBRT) is an ablative local treatment for patients with primary and secondary liver malignancies. In this setting, durable local control is increasingly relevant as improvements in systemic treatment and multimodal oncologic care have prolonged survival and increased the need for repeated local therapies over the course of disease [1]. In patients with liver metastases treated with SBRT, higher biologically effective dose (BED) has been associated with improved local hepatic control, underlining the importance of safely delivering ablative treatments whenever possible [2], [3].

A major challenge in liver SBRT is respiratory-induced target motion. In routine practice, motion is commonly addressed using an internal target volume (ITV) concept derived from four-dimensional computed tomography (4D-CT). While robust, this approach incorporates the full motion envelope into the target volume and may thereby increase the planning target volume (PTV), irradiated normal liver volume, and dose to adjacent organs at risk. This is particularly relevant in liver SBRT, where preservation of uninvolved liver parenchyma and sparing of nearby gastrointestinal structures are critical [4].

Another conventional approach for target localization is the implantation of intra- or perilesional fiducial markers. However, this is an invasive procedure that may be complicated by bleeding or marker displacement, and multiple lesions may require placement of more than one marker. In addition, fiducial implantation can delay treatment, increase costs, and provide only surrogate information on actual tumor motion [5] [6].

Magnetic resonance-guided radiotherapy (MRgRT) provides superior soft-tissue visualization and enables online adaptation and intrafraction imaging. Clinical studies have demonstrated the feasibility and safety of MR-guided SBRT for both primary and secondary liver tumors [2], [4]. More recently, intrafraction motion management in MR-guided radiotherapy has evolved from monitoring-only approaches towards active motion management, including respiratory gating. Modern MR-guided systems enable real-time target or surrogate tracking and automated beam gating based on predefined positional criteria. Early reports suggest that gating and drift compensation on the 1.5 T MR-Linac improve dosimetric accuracy for motile upper abdominal targets, while technical studies have confirmed the performance of the underlying motion-management system [7], [8]. However, evidence specific to respiratory-gated MR-guided liver SBRT using Comprehensive Motion Management (CMM) remains limited. Current reports mainly focus on technical validation, commissioning, or early implementation, whereas the dosimetric effect of introducing CMM into routine clinical liver SBRT has not been well characterized. In addition, only limited comparative data are available against established ITV-based non-gated workflows [7], [8], [9].

Here we report our experience with CMM in the first 20 patients treated with liver SBRT on a 1.5 T MR-Linac at our institution. Clinically delivered CMM plans for primary or metastatic liver malignancies were compared with retrospectively generated ITV-based plans simulating the previously used non-gated workflow using identical prescription doses. We hypothesized that CMM would reduce motion-related treatment volumes and improve sparing of uninvolved liver and other organs at risk, thereby increasing the dosimetric feasibility of ablative treatment.

## 2 Material and methods

### 2.1 Patient selection

This dosimetric analysis included the first 20 consecutive patients treated with online adaptive MR-guided SBRT for primary or secondary liver malignancies on the 1.5 T MR-Linac (Unity, Elekta AB, Stockholm, Sweden) using CMM between October 2024 and April 2025. All patients provided written informed consent for prospective clinical data collection through the MOMENTUM study (Multi-OutcoMe EvaluatioN of radiation Therapy Using the MR-Linac; ClinicalTrials.gov identifier NCT04075305). The MOMENTUM study was approved by the Ethics Committee of the Medical Faculty of the University of Tübingen (reference 059/2023BO1), and the study was conducted in accordance with the Declaration of Helsinki.

### 2.2 Simulation and imaging

A 4D-CT simulation scan in treatment position with arms above the head using indexed patient positioning aids was acquired and four respiratory phases were reconstructed. As a surrogate of respiratory motion amplitude, the superior–inferior excursion of the liver dome was measured between the extreme 4D-CT phases. In addition, multiple magnetic resonance imaging (MRI) simulation sequences were acquired on the 1.5 T MR-Linac in treatment position, including navigator-triggered T2-weighted (T2W) and spectral attenuated inversion recovery T2-weighted (T2W SPAIR) at near end-expiration, as well as b3DVane, T1 3DVane, and diffusion-weighted imaging (DWI). Detailed MRI acquisition parameters are provided in the Supplementary Material.

Before simulation and each treatment fraction, patients were instructed to fast for at least 3 h. If the target lesion was located close to gastrointestinal organs at risk, such as the bowel, stomach, or duodenum, patients additionally received scopolamine butylbromide (Buscopan®) to reduce bowel motility during imaging and treatment delivery. Intrafraction tumor motion was further passively mitigated using an abdominal compression belt.

Delineation was performed in Monaco® V. 6.2.2. For each plan, the following organs-at-risk (OARs) were delineated: uninvolved liver (liver-GTV), lungs, chest wall, kidneys, esophagus, stomach, duodenum, large and small bowel, and heart.

### 2.3 CMM and ITV treatment workflows

All patients were clinically treated using the CMM workflow on the Elekta Unity 1.5 T MR-Linac. For dosimetric comparison, a corresponding retrospective ITV-based plan was generated for each delivered CMM plan to simulate our previous non-gated liver SBRT approach.

The primary dataset for GTV delineation was the navigator-triggered T2-weighted MRI acquired near end-expiration, complemented by additional diagnostic images. This sequence was chosen because it represents the end-expiratory target position used as gating reference, whereas free-breathing radial sequences such as b3DVane represent a motion-averaged position. Acquisition took approximately 4 min, was repeated before every fraction and was successful in all patients. The PTV margins were 7 mm in the inferior and anterior directions and 5 mm in all remaining directions. As the PTV simultaneously served as the gating envelope and the GTV was defined at end-expiration, the margin was enlarged in the inferior and anterior directions to account for residual respiratory motion within the gating window [8]; the 5 mm margin in the remaining directions accounted for setup, delineation and tracking uncertainties [8], [10]. Although larger than the ITV margin in two directions, the CMM margin was applied to the GTV rather than to an ITV encompassing the full respiratory motion, resulting in smaller PTVs.

For retrospective comparison, an ITV-based plan was generated using the 4D-CT, the planning MRI, and additional diagnostic imaging for target delineation. The ITV was obtained by encompassing all respiratory phases, and the corresponding PTV was generated by adding an isotropic 5-mm margin.

In the clinical workflow, online adaptation was performed using either the adapt-to-shape (ATS) or adapt-to-position (ATP) approach, depending on geometric requirements of the individual case or whether anatomical changes relevant to target coverage or OAR sparing required recontouring and replanning. Intrafraction motion was managed with the CMM system using expiration gating linked to navigator-triggered T2-weighted imaging acquired near end-expiration. Target motion was monitored on continuous bTFE cine MR images with the anatomical position monitoring (APM) system. Target motion was determined by registration of the cine images using a registration structure consisting of a uniform expansion of the GTV intersecting the adjacent liver edge or diaphragm while avoiding nearby ribs [11]. Gating was performed using a 95% threshold, i.e., beam delivery was interrupted when less than 95% of the GTV, at its position derived from this registration, was located within the gating envelope, defined as the PTV.

For gating-efficiency analysis, the generated log files were evaluated for each treated fraction. Recorded beam-on and beam-hold intervals were then extracted to quantify delivery efficiency. Active delivery time was defined as the delivery time excluding gating-related beam holds, i.e., including beam-on time, gantry rotation and MLC segment transitions. Clinical feasibility was defined as completion of all planned fractions with the gated workflow without conversion to non-gated delivery.

### 2.4 Treatment planning

Treatment plans were generated in Monaco® V. 6.2.2 using six to nine beam angles. Plans were calculated based on the 4D CT in the reconstructed exhale phase.

Dose prescription and organ-at-risk constraints followed the institutional standard for liver SBRT, which incorporates recommendations from both the UK SABR guidelines and the Timmerman recommendations [12], [13].

Prescription doses ranged from 30 to 50 Gy delivered in 3 to 5 fractions, prescribed to the 65% or 80% isodose line.

ITV-based comparator plans were generated by one medical physicist with 10 years of experience in SBRT planning, including eight years on the MR-Linac in Monaco® V. 6.2.2 following a standardized procedure. The clinical CMM plans had been created independently by the clinical physicists, and their dosimetric results were not reviewed during replanning. The clinically approved CMM reference plan served as the starting point, and beam angles, prescription dose, isodose normalization, segmentation settings, and dose calculation parameters were kept unchanged. The PTV_CMM was replaced by the PTV_ITV in all target cost functions. After reoptimization, if PTV_ITV coverage (D98% ≥ 95% of the prescription dose) was not achieved, the OAR cost function most strongly limiting target coverage identified by its relative impact was relaxed and the plan was reoptimized. This process was repeated until coverage was restored or further relaxation would have exceeded the institutional OAR constraint. In the latter case, OAR constraints were prioritized and reduced target coverage was accepted, consistent with our clinical practice.

The assessed parameters included target volume metrics, liver dose–volume parameters, and organ-at-risk dose constraints for the uninvolved liver, right kidney, chest wall, esophagus, stomach, duodenum, large bowel, and heart. Specifically, the following parameters were analyzed: PTV volume (cc); GTV Dmean and D98%; PTV D100%, D98%, D95%, D50% and D2% (in % of the prescription dose); MU/Gy; uninvolved liver Dmean, D ≥ 700 cc, V15 (cc) and V10 (%); right kidney Dmean and V6 (%); chest wall D0.035 cc, D0.1 cc and D30 cc; large bowel D0.1 cc, D0.5 cc and D5 cc; stomach D0.1 cc, D5 cc, D10 cc and D50 cc; duodenum D0.1 cc, D5 cc and D10 cc; esophagus D0.1 cc and D5 cc; heart D0.035 cc, D0.1 cc and D15 cc (Supplementary Material Table 3).

**Table 3:** Treatment times and delivery efficiency of the clinically delivered CMM treatments by fractionation scheme. Values are plan-level medians across all evaluated fractions; group values are given as the mean of the plan-level medians.

Plan	Prescription dose (Gy)	Active delivery time (min)	Treatment time (min)	Delivery efficiency (%)	Evaluated fractions
**Three-fraction plans (*n* = 7)**					
03	45	5.1	8.4	60.5	3
10	45	8.0	10.3	78.2	3
13	30	7.5	7.8	93.6	3
14	45	9.6	21.1	41.7	3
16	36	3.9	5.4	72.4	3
17	45	9.2	24.0	40.7	3
21	45	9.8	12.8	76.9	3
**Mean**		**7.6**	**12.8**	**66.3**	**21**
**Five-fraction plans (*n* = 15)**					
01	50	4.9	11.1	49.9	5
02	40	7.0	7.5	93.6	3
04	50	8.3	8.9	92.0	5
05	35	4.2	9.6	44.7	5
06	50	5.2	9.9	58.8	5
07	40	8.8	9.0	98.4	5
08	35	7.1	8.1	90.1	5
09	35	5.3	7.1	74.2	5
11	40	7.7	11.3	65.5	5
12	50	6.6	9.9	65.5	5
15	35	7.6	12.2	59.4	5
18	50	8.2	10.7	78.5	5
19	40	7.3	8.0	91.8	5
20	50	6.5	8.0	80.5	4
22	50	5.7	8.5	66.9	5
**Mean**		**6.7**	**9.3**	**74.0**	**72**

Active delivery time, time from first beam-on to end of irradiation; treatment time, time from CMM start to end of irradiation; delivery efficiency, proportion of the active delivery time with the beam on. Evaluated fractions: fractions with available delivery log files.

Plan quality was assessed using the conformity index (CI = V_Rx / PTV volume), R50% (V_50% / PTV volume) and the gradient index (GI = V_50% / V_Rx), where V_Rx and V_50% denote the body volumes receiving at least 100% and 50% of the prescription dose.

### 2.5 Statistical analysis

The analysis was performed at the plan level, comparing 22 matched CMM and ITV plan pairs. Both plans of each pair were prescribed to the same isodose line. Continuous variables were summarized as median and range. Paired differences (ITV − CMM; positive values indicating higher values in the ITV workflow) were tested with the two-sided exact Wilcoxon signed-rank test. Effect sizes were reported as Hodges–Lehmann estimate of the paired difference with 95% confidence interval and as matched-pairs rank-biserial correlation (r_rb_). Target dose parameters (normalized to the prescription dose) and plan-quality indices (CI, R50%, GI, V50%, MU/Gy) were analyzed across all plans (*n* = 22); OAR parameters were analyzed separately for three- (*n* = 7) and five-fraction plans (*n* = 15). *P*-values were adjusted with the Benjamini–Hochberg procedure within each of these four groups, and adjusted *p*-values <0.05 were considered significant. As no DVH parameter was predefined as primary endpoint, the DVH analysis was considered exploratory. Violations unique to one workflow were compared using the exact McNemar test. Statistical analyses were performed in Python using the SciPy library.

## 3 Results

### 3.1 Patient and treatment characteristics

Twenty patients were included, seven female, thirteen male. Mean age at treatment was 65 (range 44—90). Five patients had two treated lesions; in two of these patients, the lesions were treated with separate isocenters, resulting in two independent plans, whereas in three patients both lesions were treated within a single-isocenter plan. Accordingly, the primary dosimetric analysis was performed at the plan level, comparing 22 matched CMM and ITV plan pairs. Fourteen patients (70%) were treated for metastatic disease, six (30%) for hepatocellular carcinoma. Two patients previously had received liver SBRT for another lesion, six had undergone liver surgery prior to SBRT. Five lesions were treated with 45 Gy in 3 fractions (prescribed to an isodose level, IDL, of 65%), one with 36 Gy in 3 fractions (IDL 80%), one with 30 Gy in 3 fractions (IDL 80%), seven with 50 Gy in 5 fractions (IDL 80%), four lesions with 40 Gy in 5 fractions (IDL 80%), four with 35 Gy in 5 fractions (IDL 80%) (Supplementary Material Table 2). The median superior–inferior liver dome excursion on 4D-CT was 11.1 mm (range 3.5–23.6 mm). Liver dome excursion correlated with the relative PTV reduction (ρ = 0.58, *p* = 0.005), but not with the reduction in liver-GTV Dmean (ρ = −0.14, *p* = 0.55) or the number of resolved OAR violations (ρ = −0.27, *p* = 0.22).

### 3.2 Dosimetric comparison of CMM and ITV plans

Median PTV volume for CMM plans was 28.0 [6.2—125.2] cc. Median PTV volume for ITV plans was 41.9 [7.5—165.8] cc, with a median volume difference between plans of 10.1 [1.3—40.7] cc (Hodges–Lehmann estimate 13.3 cc, 95% confidence interval 7.9–20.2). The mean uninvolved liver volume over all patients was 1473.8 [1041.5—1935.4] cc. Median monitor units (MU) per fraction of the reference plans were 1784.0 [1052.1—2898.6] and for ITV plans 1889.1 [991.2—2731.5]. Differences in the MU efficiency (MU/Gy) were not significant, with a median difference between workflows of −0.05 MU/Gy (*p* = 0.50). MU efficiency was evaluated as a surrogate of plan complexity and beam-on time; thus, the smaller CMM target volumes did not require increased plan modulation. CI and R50% were slightly higher with CMM (median 1.15 vs. 1.13 and 6.00 vs. 5.83; adjusted *p* = 0.070 each), whereas the absolute volume receiving ≥50% of the prescription dose was lower with CMM in all 22 plans (adjusted *p* < 0.001).

Different OAR constraints were applied for three and five fractions, respectively (Supplementary Material Table 3). In the OAR evaluation, the CMM workflow resulted in 10 constraint violations, whereas the ITV-based workflow resulted in 19 violations. Details of all violations are shown in Table 2.

At the plan level, at least one OAR constraint violation occurred in 8 of 22 CMM plans (36%) and in 12 of 22 ITV plans (55%). Considering both OAR constraints and target coverage (PTV D98% ≥ 95%), 14 CMM plans (64%) and 9 ITV plans (41%) fulfilled all planning criteria. Violations unique to one workflow were more frequent with ITV (11 vs. 2, exact McNemar test, *p* = 0.022, odds ratio 5.5, 95% CI 1.2–51.1).

With the CMM approach one violation of the desired target coverage, defined as PTV D98% < 95% was noted against four violations with the ITV approach. However, the Wilcoxon signed-rank test showed no significant difference in PTV D98% coverage between workflows (*p* = 0.41). Significant differences were observed for PTV D100%, D50%, and D2%, with median differences of −2.1%, −1.8% and − 0.7% of the prescription dose, respectively, indicating higher values in the CMM workflow. Detailed results are provided in Table 1.

**Table 1:** Paired differences in dose–volume parameters between ITV-based and CMM plans, shown for all parameters with a nominal *p*-value below 0.05 in the Wilcoxon signed-rank test. Differences are given as ITV − CMM; positive values indicate higher values in the ITV workflow. Target dose parameters are expressed as percentage of the prescription dose. For each parameter, the reported *p*-value is from the two-sided Wilcoxon signed-rank test; the adjusted *p*-value was additionally calculated using the Benjamini–Hochberg procedure, applied separately within four groups of comparisons: target volume dose (*n* = 22, 8 tests), plan quality (*n* = 22, 5 tests), three-fraction OAR parameters (*n* = 7, 24 tests), and five-fraction OAR parameters (*n* = 15, 24 tests). Effect sizes are given as Hodges–Lehmann (HL) estimate of the paired difference with 95% confidence interval (not adjusted for multiple testing) and as rank-biserial correlation (r_rb_; ±1, all pairs differ in the same direction).

Parameter	n	Median difference ITV − CMM [range]	HL estimate [95% confidence Interval]	r_rb_	*p*-value	adjusted p-Value
**All plans**						
*Target volume and dose*						
PTV volume (cc)	22	10.11 [1.31, 40.68]	13.32 [7.88, 20.15]	1.00	< 0.001	<0.001
PTV D100% (%)	22	−2.05 [−45.37, 5.70]	−2.08 [−7.04, −0.50]	−0.61	0.010	0.020
PTV D50% (%)	22	−1.75 [−11.20, 2.08]	−1.73 [−2.63, −0.81]	−0.77	< 0.001	< 0.001
PTV D2% (%)	22	−0.67 [−10.63, 4.44]	−0.95 [−2.24, −0.23]	−0.61	0.010	0.020
**Plan quality**						
CI	22	−0.03 [−0.58, 0.11]	−0.03 [−0.06, −0.01]	−0.50	0.039	0.070
R50%	22	−0.31 [−1.93, 0.84]	−0.30 [−0.61, −0.01]	−0.49	0.042	0.070
V50% (cc)	22	54.0 [5.4, 269.2]	68.2 [40.6, 106.9]	1.00	< 0.001	< 0.001
**Three-fraction plans**						
Liver-GTV Dmean (Gy)	7	0.97 [0.25, 2.12]	0.97 [0.35, 1.70]	1.00	0.016	0.037
Liver D ≥ 700 cc (Gy)	7	0.54 [0.03, 0.94]	0.36 [0.08, 0.77]	1.00	0.016	0.037
Liver-GTV V15Gy (cc)	7	31.50 [7.89, 115.0]	31.81 [11.06, 77.74]	1.00	0.016	0.037
Liver-GTV V10Gy (%)	7	3.32 [0.44, 7.34]	3.43 [0.73, 7.18]	1.00	0.016	0.037
Kidney R Dmean (Gy)	7	0.08 [0.00, 1.41]	0.11 [0.01, 0.77]	1.00	0.031	0.058
Chest wall D30cc (Gy)	7	1.08 [0.45, 3.25]	1.09 [0.63, 2.22]	1.00	0.016	0.037
Bowel D0.5cc (Gy)	7	0.59 [−0.11, 5.24]	0.70 [0.01, 2.99]	0.86	0.047	0.075
Bowel D5cc (Gy)	7	0.77 [0.00, 5.03]	0.84 [0.07, 2.95]	1.00	0.031	0.058
Stomach D5cc (Gy)	7	0.41 [0.05, 6.16]	0.41 [0.06, 3.31]	1.00	0.016	0.037
Stomach D10cc (Gy)	7	0.52 [0.05, 5.00]	0.54 [0.16, 2.80]	1.00	0.016	0.037
Stomach D50cc (Gy)	7	0.48 [0.03, 1.47]	0.46 [0.04, 1.08]	1.00	0.016	0.037
Duodenum D0.1cc (Gy)	7	0.24 [0.00, 4.65]	0.42 [0.06, 2.57]	1.00	0.031	0.058
Heart D0.035cc (Gy)	7	0.10 [0.02, 0.59]	0.10 [0.03, 0.44]	1.00	0.016	0.037
Heart D0.1cc (Gy)	7	0.07 [0.01, 0.65]	0.09 [0.03, 0.58]	1.00	0.016	0.037
Heart D15cc (Gy)	7	0.03 [−0.02, 0.33]	0.07 [0.01, 0.21]	0.86	0.047	0.075
**Five-fraction plans**						
Liver-GTV Dmean (Gy)	15	0.93 [−0.94, 3.75]	1.08 [0.60, 1.59]	0.87	0.002	0.009
Liver D ≥ 700 cc (Gy)	15	0.55 [0.05, 2.84]	0.70 [0.42, 1.21]	1.00	< 0.001	<0.001
Liver-GTV V15Gy (cc)	15	26.94 [7.90, 149.9]	33.72 [20.80, 68.59]	1.00	< 0.001	<0.001
Liver-GTV V10Gy (%)	15	3.98 [−3.82, 9.22]	3.82 [1.69, 5.49]	0.88	0.001	0.004
Kidney R Dmean (Gy)	15	0.14 [0.02, 8.75]	0.28 [0.10, 2.01]	1.00	< 0.001	<0.001
Kidney R V6Gy (%)	15	1.00 [0.00, 22.06]	0.92 [0.12, 4.91]	1.00	0.008	0.018
Chest wall D30cc (Gy)	15	1.28 [0.01, 9.97]	1.32 [0.70, 2.47]	1.00	< 0.001	0.003
Bowel D0.5cc (Gy)	15	0.50 [−1.35, 13.96]	0.67 [0.08, 4.17]	0.62	0.035	0.065
Stomach D50cc (Gy)	15	0.49 [−0.66, 1.39]	0.45 [0.21, 0.72]	0.83	0.006	0.017
Duodenum D0.1cc (Gy)	15	0.20 [−1.49, 12.33]	0.37 [0.04, 2.50]	0.60	0.041	0.071
Duodenum D5cc (Gy)	15	0.23 [0.01, 12.17]	0.79 [0.15, 3.75]	1.00	< 0.001	0.003
Duodenum D10cc (Gy)	15	0.21 [0.01, 3.54]	0.39 [0.13, 1.24]	1.00	< 0.001	<0.001
Heart D0.035cc (Gy)	15	0.08 [−0.22, 5.76]	0.38 [0.03, 0.96]	0.67	0.022	0.046
Heart D15cc (Gy)	15	0.07 [−0.23, 3.55]	0.12 [0.01, 0.78]	0.71	0.023	0.046

n, number of evaluated plans; CI, conformity index (V_Rx / PTV volume); R50%, V_50% / PTV volume; GI, gradient index (V_50% / V_Rx); V_Rx and V_50%, body volumes receiving at least 100% and 50% of the prescription dose; MU, monitor units; Liver-GTV, uninvolved liver; R, right.

All four significant target dose and volume differences remained significant after correction (adjusted *p* ≤ 0.020). Among plan-quality and OAR parameters, 23 of 32 nominally significant differences remained significant; nine borderline differences lost significance (adjusted *p* = 0.058–0.075; Table 1). For the majority of evaluated parameters, the CMM-based workflow showed greater median organ sparing. In the five-fraction treatments, parameters with *p*-values below 0.01 included Liver-GTV Dmean, with a median difference of 0.93 [−0.94—3.75] Gy (Hodges–Lehmann estimate 1.08 Gy, 95% confidence interval 0.60–1.59); liver dose to 700 cc, with a median difference of 0.55 [0.05—2.84] Gy; Liver-GTV V15Gy, with a median difference of 26.94 [7.90—149.9] cc; Liver-GTV V10Gy, with a median difference of 3.98 [−3.82—9.22] %; kidney right Dmean, with a median difference of 0.14 [0.02—8.75] Gy; kidney right V6Gy, with a median difference of 1.00 [0—22.06] %; chest wall D30cc, with a median difference of 1.28 [0.01—9.97] Gy; stomach D50cc, with a median difference of 0.49 [−0.66—1.39] Gy; duodenum D5cc, with a median difference of 0.23 [0.01—12.17] Gy; and duodenum D10cc, with a median difference of 0.21 [0.01—3.54] Gy. In the three-fraction subgroup, the lowest observed *p*-value was 0.016, which was attributable to the limited sample size of seven paired plans. Additional details, including effect sizes, are provided in Table 1 and in the supplementary table 4, together with a visual representation of the absolute values and workflow-related differences in Fig. 1. An example of the dose distributions of both workflows is shown in Fig. 2.

**Fig. 1:**
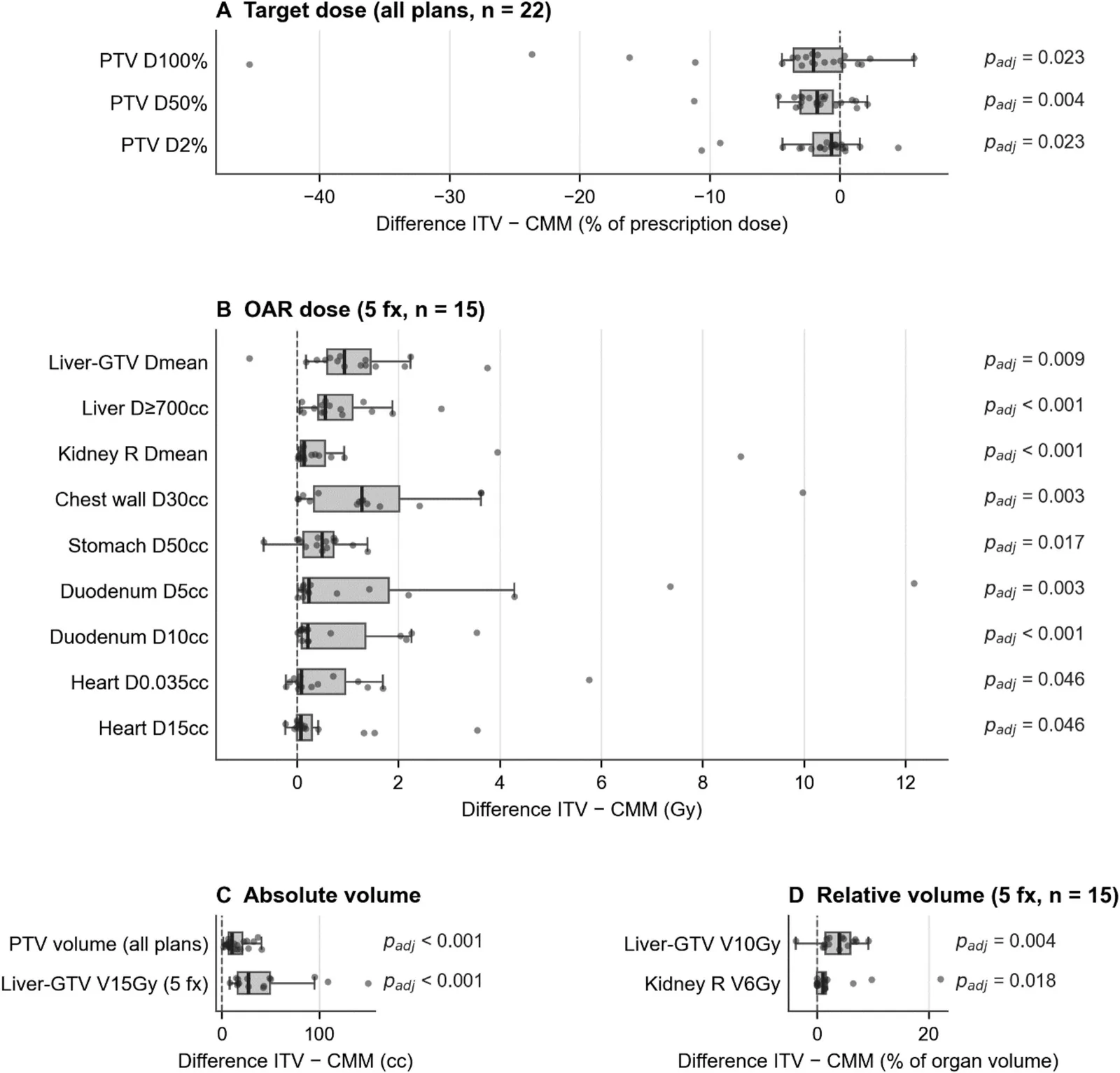
Paired differences in dose–volume parameters between ITV-based and CMM plans, grouped by metric type. (A) Target dose, expressed as percentage of the prescription dose, across all plans (*n* = 22). (B) Organ-at-risk dose in five-fraction plans (*n* = 15). (C) Absolute volume metrics. (D) Relative volume metrics in five-fraction plans (*n* = 15). Differences are given as ITV − CMM; positive values indicate higher values in the ITV workflow. Only parameters with a Benjamini–Hochberg adjusted *p*-value (p_adj_) below 0.05 are shown.

**Fig. 2:**
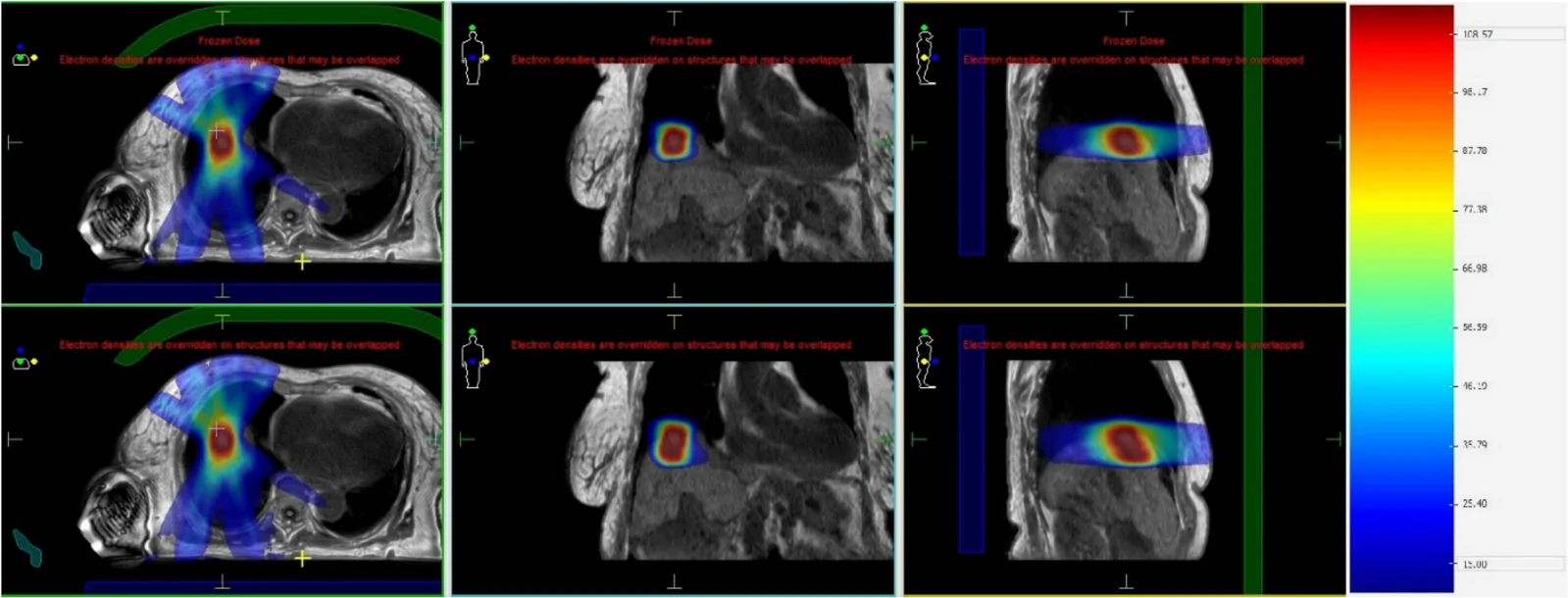
Qualitative illustration of the isodose distribution for an SBRT plan delivering 36 Gy in 3 fractions. The upper row shows the CMM-based plan and the lower row the ITV-based plan, each in axial, coronal and sagittal views. The color scale on the right indicates the dose as percentage of the prescription dose. This case showed the largest relative PTV-volume difference, with PTV volumes of 17.39 cc (ITV) and 9.25 cc (CMM), corresponding to a 46.8% reduction.

Due to log-file corruption, 3 of 96 treatments were not available for evaluation. Group values are reported in Table 3 as the mean of the plan-level median delivery efficiency as well as irradiation and treatment times. For three-fraction treatments, the mean of the plan-level median active delivery time was 7.6 min and the mean treatment time from CMM start to the end of irradiation was 12.8 min, with a mean delivery efficiency of 66.3% and plan-level efficiencies ranging from 40.7% to 93.6%. For five-fraction treatments, which were delivered with a lower dose per fraction, the corresponding values were 6.7 min, 9.3 min, and 74.0%, with plan-level efficiencies ranging from 44.7% to 98.4% (Table 3).

## 4 Discussion

In this initial clinical experience, implementation of Comprehensive Motion Management (CMM) for liver SBRT on a 1.5 T MR-Linac resulted in smaller motion-related target volumes and improved organ-at-risk sparing compared with a retrospectively simulated ITV-based non-gated workflow, while maintaining comparable target coverage. At the same time, treatment delivery remained clinically feasible, with all fractions completed and acceptable irradiation and treatment times. Taken together, these findings suggest that expiration-gated CMM can translate the theoretical advantages of MR-guided intrafraction motion management into practical dosimetric benefit in routine liver SBRT [7].

The most direct finding of the present study is the reduction in PTV associated with the CMM workflow. This is clinically intuitive, as CMM defines the target in a reproducible end-expiratory state and manages residual motion during delivery through gating, rather than incorporating the full respiratory motion envelope into the target volume. In the liver, where sparing of gastrointestinal organs and uninvolved parenchyma is essential, even moderate reductions in PTV may have meaningful dosimetric consequences. In our cohort, smaller target volumes with CMM were accompanied by fewer OAR constraint violations and improved normal tissue sparing. Importantly, better preservation of uninvolved liver may also help maintain the option of future retreatment, which is increasingly relevant as advances in systemic therapy, surgery, and radiotherapy prolong survival and make repeated local ablative interventions more feasible.

The clinical relevance of these dosimetric differences should be interpreted with caution. For several parameters, the median absolute reductions were small and are unlikely to be of clinical consequence for the average patient. For example, right kidney Dmean, duodenum D10cc and heart D15cc showed a consistent direction (r_rb_ 0.71–1.00) but small Hodges–Lehmann estimates (0.12–0.39 Gy). However, the distribution of differences was markedly skewed: while most plan pairs showed modest differences, individual cases showed substantial reductions of up to 8.8 Gy in right kidney Dmean, 150 cc in liver-GTV V15Gy and 12.2 Gy in duodenum D5cc (Table 1). The dosimetric benefit of CMM therefore appears to be concentrated in a subset of patients, presumably those with lesions in close proximity to radiosensitive OARs, which is in line with the question of patient selection raised by Hoegen-Saßmannshausen et al. [9]. With respect to toxicity, the reductions in near-maximum doses to the luminal gastrointestinal organs are likely the most relevant, as these serial organs exhibit a steep dose–response relationship for ulceration, bleeding and perforation, so that small dose reductions close to the tolerance limit may substantially alter complication risk [14], [15]. In contrast, the median reduction in liver-GTV Dmean of approximately 1 Gy is unlikely to influence the risk of radiation-induced liver disease after a single treatment course in patients with adequate liver reserve, as all liver constraints were met with both workflows. Its relevance lies rather in patients with compromised liver function, such as those with cirrhosis-associated hepatocellular carcinoma, and in the context of cumulative dose from repeated treatments [16], [17].

These findings are consistent with the broader rationale of MR-guided liver SBRT. Prospective and retrospective MR-guided liver series have shown that MRgRT is feasible and safe for both primary and metastatic liver tumors, with favorable toxicity profiles and promising local control. In particular, van Dams et al. reported that ablative MR-guided SBRT for primary and secondary liver tumors was viable and safe, while Weykamp et al. demonstrated encouraging long-term outcomes for liver metastases and noted improved local control with BED >100 Gy, emphasizing the importance of safely delivering highly ablative treatment [2], [4]. The present analysis does not itself demonstrate improved local control, but it supports one of the key prerequisites for dose intensification: reduced treatment volumes and lower exposure of uninvolved liver and adjacent OARs.

An important strength of this study is that it adds comparative clinical data to the still limited literature on CMM-based liver SBRT. Grimbergen et al. demonstrated dosimetric benefits of gating and intrafraction drift correction for upper abdominal tumors on a 1.5 T MR-Linac [7]. By comparing clinically delivered CMM plans with retrospectively generated ITV-based plans reflecting our former institutional approach, the present analysis provides practical evidence for the dosimetric impact of CMM in routine liver SBRT. Our findings are in line with the recent comparative planning data by Hoegen-Saßmannshausen et al. and add complementary information from routine clinical practice [9]. While their study focused on hepatic metastases, our cohort also included primary liver malignancies and reflects the clinical implementation of expiration-gated CMM on a 1.5 T MR-Linac. In addition, we combined the dosimetric comparison with early workflow feasibility data.

Another important finding is the reduction in OAR constraint violations, which is arguably more clinically meaningful than differences in individual DVH parameters, as institutional constraints define the threshold at which planning compromises become necessary. The number of constraint violations was nearly halved with CMM (10 vs. 19), and the proportion of plans fulfilling all OAR constraints and the target coverage criterion increased from 41% to 64%. In addition, the ITV approach more frequently required a compromise in target coverage: four ITV plans compared with one CMM plan did not reach PTV D98% ≥ 95%. In two of these cases, PTV_ITV D98% decreased to 75.4% and 82.3% of the prescription dose, whereas full coverage was achieved with CMM (Table 2). In clinical practice, such cases would have required either acceptance of relevant target underdosage or dose de-escalation. However, constraint violations were not eliminated with CMM. Four of the ten remaining violations involved the chest wall, five concerned near-maximum doses to gastrointestinal OARs directly adjacent to the PTV, and one the heart. It should be noted that this comparison was based on treatment-planning dose distributions and did not account for time-resolved dose delivery; intrafraction motion and gating dynamics may therefore result in differences between planned and actually delivered dose. Nevertheless, the planning comparison suggests that active motion management may help maintain the balance between target coverage and normal tissue protection in anatomically challenging cases.

**Table 2:** Target coverage and organ-at-risk (OAR) constraint violations according to the institutional liver SBRT standard based on UK SABR consensus and Timmerman recommendations. Only plans and parameters with a violation in at least one workflow are shown.

Plan	Fx	Parameter	Limit	ITV	CMM	Status
**Target coverage**						
05	5	PTV D98% (%)	≥ 95	94.83	96.94	Resolved
08	5	PTV D98% (%)	≥ 95	75.40	100.9	Resolved
09	5	PTV D98% (%)	≥ 95	82.29	99.83	Resolved
13	3	PTV D98% (%)	≥ 95	91.90	93.37	Persisting
**OAR constraints**						
01	5	Kidney R Dmean (Gy)	≤ 10	15.32	6.57	Resolved
01	5	Kidney R V6Gy (%)	≤ 30	39.12	17.06	Resolved
04	5	Esophagus D0.1cc (Gy)	≤ 35	36.03	27.01	Resolved
09	5	Bowel D5cc (Gy)	≤ 30	30.28	20.07	Resolved
11	5	Stomach D0.1cc (Gy)	≤ 33	33.30	30.35	Resolved
11	5	Heart D0.1cc (Gy)	≤ 35	36.35	34.75	Resolved
13	3	Stomach D10cc (Gy)	≤ 16.5	17.99	12.99	Resolved
19	5	Bowel D0.5cc (Gy)	≤ 33	41.11	32.87	Resolved
19	5	Bowel D5cc (Gy)	≤ 30	35.13	22.27	Resolved
19	5	Duodenum D5cc (Gy)	≤ 26.5	26.80	24.60	Resolved
21	3	Chest wall D0.1cc (Gy)	≤ 36.9	38.33	34.90	Resolved
06	5	Chest wall D0.1cc (Gy)	≤ 43	54.78	52.51	Persisting
08	5	Stomach D50cc (Gy)	≤ 12	13.52	12.95	Persisting
09	5	Bowel D0.5cc (Gy)	≤ 33	34.57	33.59	Persisting
13	3	Stomach D0.1cc (Gy)	≤ 22.2	28.19	27.72	Persisting
14	3	Chest wall D0.1cc (Gy)	≤ 36.9	47.58	49.43	Persisting
15	5	Heart D0.1cc (Gy)	≤ 35	36.08	36.34	Persisting
19	5	Bowel D0.1cc (Gy)	≤ 36	44.94	45.46	Persisting
20	5	Chest wall D0.1cc (Gy)	≤ 43	54.35	52.67	Persisting
14	3	Chest wall D0.035cc (Gy)	≤ 50	47.91	50.15	New with CMM
19	5	Duodenum D0.1cc (Gy)	≤ 33	32.70	34.32	New with CMM
**Total OAR constraint violations**				**19**	**10**	
**Plans with PTV D98% < 95%**				**4**	**1**	
**Plans with ≥ 1 OAR constraint violation**				**12**	**8**	
OAR violations with ITV resolved by CMM						**11 of 19**
OAR violations persisting in both workflows						**8 of 19**
New OAR violations with CMM only						**2**
PTV coverage failures with ITV resolved by CMM						**3 of 4**

Fx, number of fractions; ITV, internal target volume-based plan; CMM, Comprehensive Motion Management plan. Status compares the ITV and CMM plan of the same case for the same parameter: resolved = constraint violated with ITV but met with CMM; persisting = constraint violated with both workflows; new with CMM = constraint met with ITV but violated with CMM.

This is particularly relevant in liver SBRT because long-term disease trajectories are changing. With improving systemic therapy and increasing use of repeated local ablative interventions, re-irradiation or treatment of additional hepatic lesions is becoming increasingly realistic [18], [19]. In addition, liver SBRT is increasingly used as bridging therapy in selected patients with hepatocellular carcinoma awaiting liver transplantation, further highlighting the importance of preserving uninvolved liver parenchyma [20], [21], [22]. In this setting, sparing uninvolved liver is not merely a dosimetric endpoint but a way of preserving future treatment options. Although our study does not directly evaluate retreatment feasibility or cumulative liver tolerance, the observed reductions in uninvolved liver dose metrics and OAR constraint violations support the hypothesis that CMM may improve the dosimetric feasibility of repeated or sequential local treatment strategies. Beyond retreatment, the dosimetric gains may also facilitate dose escalation. Higher BED has been associated with improved local control of liver metastases, with a BED >100 Gy proposed as a relevant threshold [2], [3]. In our cohort, this benefit was most evident in the cases in which ITV-based planning required substantial coverage compromise, whereas CMM allowed delivery of the intended prescription. In the remaining plans, the OAR dose reductions achieved with CMM might provide additional headroom for dose intensification. However, whether this translates into higher achievable prescription doses was not evaluated in the present study and should be addressed in dedicated planning studies.

The delivery data add an important practical dimension to the dosimetric findings. One common concern regarding gated MR-guided workflows is that improved precision may come at the cost of treatment efficiency or patient tolerance. In our cohort, however, treatment times remained within a clinically acceptable range, and delivery efficiencies were compatible with routine use. These findings support the practical feasibility of expiration-gated MR-guided liver SBRT in routine clinical practice.

Several limitations should be acknowledged. First, this was a single-institution retrospective dosimetric comparison with a small sample size. The number of available plan pairs was limited, particularly after subdivision into three- and five-fraction treatments, and this reduces statistical power for some subgroup analyses. Given the number of dosimetric endpoints, type I error cannot be excluded. Although all comparisons were corrected for multiple testing and all target-related differences persisted, several OAR differences lost significance after adjustment and should be regarded as hypothesis-generating. Second, the comparator plans were retrospectively generated rather than clinically delivered. Although this paired design is a strength in that it allows within-case comparison using identical prescription dose, retrospective replanning cannot fully reproduce all aspects of historical real-world treatment decision-making. Moreover, the ITV plans were generated by a single planner and reused the beam geometry of the clinical plans, which served as the starting point, so that full blinding was not possible, although their dosimetric results were not reviewed during replanning. Potential bias may act in both directions: because the ITV plans were derived from plans optimized for a smaller target, the optimization strategy may not have been fully tailored to the larger PTV_ITV, potentially favoring CMM; conversely, ITV replanning was performed without the time constraints of clinical planning, potentially favoring the ITV plans. The standardized replanning procedure was intended to minimize, but cannot fully exclude, planner-dependent effects. The study therefore evaluates the dosimetric impact of introducing CMM within a fixed institutional planning framework, rather than comparing optimally individualized CMM and ITV workflows. Third, the cohort was heterogeneous, including both hepatocellular carcinoma and liver metastases, a range of lesion sizes, and different prescription doses and fractionation schedules. While this heterogeneity reflects routine clinical practice, it limits disease-specific interpretation. Owing to the small sample size, no formal subgroup analyses were performed; larger motion was associated with a greater relative PTV reduction, whereas the dosimetric benefit appeared to depend mainly on lesion proximity to gastrointestinal OARs. Motion was only approximated by liver dome excursion; a dedicated analysis of cine-MR-derived GTV motion is planned. Fourth, the analysis focused on dosimetry and workflow and does not provide clinical endpoint data such as local control, toxicity, or quality of life. Most importantly, the comparison was limited to planned dose distributions and therefore cannot capture the improved delivery accuracy that constitutes one of the main advantages of gating. Conversely, residual motion within the gating window, beam-hold latency and anatomical changes during delivery may cause the delivered dose of both workflows to deviate from the planned dose. Motion-inclusive dose reconstruction and accumulation based on the recorded cine-MR motion data and delivery log files, as performed by Grimbergen et al. for upper abdominal tumors [7], will be required to quantify the actual dosimetric benefit of CMM. Likewise, the geometric correspondence between the reference and cine bTFE images, as well as the relationship between the registration structure and the target position, was not quantitatively assessed, and the CMM margins were not derived from patient-specific log-file and cine data. Reported times refer to delivery only and do not include the adaptive workflow. Finally, the study reflects an early institutional experience, and a learning-curve effect cannot be excluded.

These limitations are balanced by several strengths. The study reflects routine clinical implementation rather than a purely technical analysis. All patients were treated according to the same institutional CMM concept using expiration gating on a 1.5 T MR-Linac, and the comparison was performed on matched plan pairs derived from the same patients and prescription schemes. By integrating geometric, dosimetric, and workflow data, the study provides a more complete picture of early CMM use in liver SBRT.

In conclusion, this study shows that implementation of expiration-gated CMM for liver SBRT on a 1.5 T MR-Linac can reduce motion-related treatment volumes and improve OAR sparing compared with a retrospectively simulated ITV-based non-gated workflow, while maintaining comparable target coverage and clinically feasible delivery times. These findings support the dosimetric and practical value of CMM in routine liver SBRT and provide an early clinical benchmark for future studies evaluating delivered-dose reconstruction, toxicity, local control, dose escalation, and retreatment feasibility.

The authors declare the following financial interests/personal relationships which may be considered as potential competing interests: Funding: DFG (ZI 736/2–1). COI: research collaborations Elekta AB, Philips, PTW Freiburg, Dr. Sennewald Medizintechnik, Kaiku Health, TheraPanacea, ITV, Brainlab. S. Boeke received funding by Merck (speaker fees, travel expenses), Elekta (speaker fees, travel expenses) and Regeneron (speaker fees).

## CRediT authorship contribution statement

**Monica Lo Russo:** Writing – review & editing, Writing – original draft, Visualization, Validation, Supervision, Software, Resources, Project administration, Methodology, Investigation, Formal analysis, Data curation, Conceptualization. **Simon Böke:** Writing – review & editing, Writing – original draft, Visualization, Validation, Supervision, Software, Resources, Project administration, Methodology, Investigation, Formal analysis, Data curation, Conceptualization. **David Mönnich:** Writing – review & editing, Writing – original draft, Visualization, Validation, Supervision, Software, Resources, Project administration, Methodology, Investigation, Formal analysis, Data curation, Conceptualization. **Moritz Schneider:** Writing – review & editing, Writing – original draft, Visualization, Validation, Supervision, Software, Resources, Project administration, Methodology, Investigation, Formal analysis, Data curation, Conceptualization. **Elgin Hoffmann:** Writing – review & editing, Writing – original draft, Visualization, Validation, Supervision, Software, Resources, Project administration, Methodology, Investigation, Formal analysis, Data curation, Conceptualization. **Jessica Boldt:** Writing – review & editing, Writing – original draft, Visualization, Validation, Supervision, Software, Resources, Project administration, Methodology, Investigation, Formal analysis, Data curation, Conceptualization. **Hathal Haddad:** Writing – review & editing, Writing – original draft, Visualization, Validation, Supervision, Software, Resources, Project administration, Methodology, Investigation, Formal analysis, Data curation, Conceptualization. **Maximilian Niyazi:** Writing – review & editing, Writing – original draft, Visualization, Validation, Supervision, Software, Resources, Project administration, Methodology, Investigation, Formal analysis, Data curation, Conceptualization. **Daniela Thorwarth:** Writing – review & editing, Writing – original draft, Visualization, Validation, Supervision, Software, Resources, Project administration, Methodology, Investigation, Formal analysis, Data curation, Conceptualization. **Cihan Gani:** Writing – review & editing, Writing – original draft, Visualization, Validation, Supervision, Software, Resources, Project administration, Methodology, Investigation, Formal analysis, Data curation, Conceptualization. **Marcel Nachbar:** Writing – review & editing, Writing – original draft, Visualization, Validation, Supervision, Software, Resources, Project administration, Methodology, Investigation, Formal analysis, Data curation, Conceptualization.

## Declaration of competing interest

The authors declare the following financial interests/personal relationships which may be considered as potential competing interests: The authors declare the following financial interests/personal relationships which may be considered as potential competing interests: Funding: DFG (ZI 736/2-1). COI: research collaborations Elekta AB, Philips, PTW Freiburg, Dr. Sennewald Medizintechnik, Kaiku Health, TheraPanacea, ITV, Brainlab. S. Boeke received funding by Merck (speaker fees, travel expenses), Elekta (speaker fees, travel expenses) and Regeneron (speaker fees).
