# Supplementary material for: Comprehensive motion management for liver SBRT on a 1.5 T MR-Linac: Initial clinical experience and dosimetric comparison with an ITV-based workflow
Source: Clin Transl Radiat Oncol. 2026 Oct 24;62:101335. doi: 10.1016/j.ctro.2026.101335 (PMC13737418; doi:10.1016/j.ctro.2026.101335)
Supplement: Supplementary file 1 [file mmc1.docx]

Supplementary Table 1. MRI sequence parameters

|  | **Navigated T2w** | **Navigated T2w SPAIR** | **B3DVane** | **T1w 3DVane** | **Cine MRI** |
| --- | --- | --- | --- | --- | --- |
| Scanning geometry | 3D | 3D | 3D | 3D | 2D |
| FOV (mm) | 320 x 448 x 240 | 340 x 457 x 300 | 500 x 500 x 220 | 500 x 500 x 220 | 425 x 425 |
| Slice thickness (mm) | 2,4 | 2,4 | 3 | 3 | 5 |
| TE/TR (ms) | 206 / 2100 | 249 / 2100 | 1,31 / 3,3 | 1,18 / 3,9 | 1,87 / 3,7 |
| Acquisition Time (min) | 4:06 | 3:03 | 4:07 | 6:01 | - |

FOV: field of view; TE/TR: Echo Time/Repetition Time

Supplementary Table 2. Patient, lesion, and treatment characteristics

| **Characteristic** | **Value** |
| --- | --- |
| Patients, n | 20 |
| Treated lesions, n | 25 |
| Matched plan pairs, n | 22 |
| Mean age, years | 65.1 (44 - 90) |
| Sex, n (%) |  |
| Female | 7 (35) |
| Male | 13 (65) |
| Diagnosis, n (%) |  |
| Primary liver malignancy | 6 (30) |
| Liver metastases | 14 (70) |
| Primary tumor type, n (%) |  |
| Hepatocellular carcinoma | 6 (30) |
| Colorectal cancer | 4 (20) |
| Cholangiocarcinoma | 3 (15) |
| Neuroendocrine tumor | 3 (15) |
| Pancreatic cancer | 2 (10) |
| NSCLC | 1 (5) |
| Head-and-neck cancer | 1 (5) |
| Previous hepatic therapy, n (%)* |  |
| SBRT | 2 (10) |
| Surgery | 6 (30) |
| TACE or RFA | 2 (10) |
| Lesions per patient, n (%) |  |
| 1 lesion | 15 (75) |
| 2 lesions | 5 (25) |
| Prescription dose, Gy |  |
| 45 Gy in 3 fractions | 5 (22.7) |
| 36 Gy in 3 fractions | 1 (4.5) |
| 30 Gy in 3 fractions | 1 (4.5) |
| 50 Gy in 5 fractions | 7 (31.8) |
| 40 Gy in 5 fractions | 4 (18.2) |
| 35 Gy in 5 fractions | 4 (18.2) |

*SBRT (stereotactic body radiation therapy), TACE (Transcatheter arterial chemoembolization), RFA (Radiofrequency ablation)

Supplementary Table 3. Fractionation-specific organ-at-risk dose constraints used for liver SBRT planning

| **Organ at risk** | **Dosimetric parameter** | **3 fractions** | **5 fractions** |
| --- | --- | --- | --- |
| Liver (uninvolved liver / liver-GTV) | D_mean_ (Gy) | 13 | 15 |
|  | Spared liver volume | ≥700 cc <15 Gy | ≥700 cc <21 Gy |
|  | V10 Gy (%) | - | 70 |
| Chest wall | D0.035 cc (Gy) | 50 | 57 |
|  | D0.1 cc (Gy) | 36.9 | 43 |
|  | D30 cc (Gy) | 30 | - |
| Right kidney | D_mean_ (Gy) | 8.5 | 10 |
|  | V6 Gy (%)* | 30 | 30 |
| Esophagus | D0.1 cc (Gy) | 25.2 | 35 |
|  | D5 cc (Gy) | 27.9 | 32.5 |
| Stomach | D0.1 cc (Gy) | 22.2 | 33 |
|  | D5 cc (Gy) | - | 26.5 |
|  | D10 cc (Gy) | 16.5 | 25 |
|  | D50 cc (Gy) | - | 12 |
| Duodenum | D0.1 cc (Gy) | 22.2 | 33 |
|  | D5 cc (Gy) | - | 26.5 |
|  | D10 cc (Gy) | 11.4 | 25 |
| Bowel | D0.1 cc (Gy) | 28.2 | 36 |
|  | D0.5 cc (Gy) | - | 33 |
|  | D5 cc (Gy) | 17.7 | 30 |
| Heart | D0.035 cc (Gy) | 30 | 38 |
|  | D0.1 cc (Gy) | 26 | 35 |
|  | D15 cc (Gy) | 24 | 32 |

*Applied when mean dose to the other kidney exceeded 18 Gy.

Footnote: Constraints were applied according to the institutional liver SBRT standard, integrating recommendations from the UK SABR guidelines and Timmerman recommendations.

Supplementary Table 4. Paired differences in all analyzed parameters between ITV-based and CMM plans. Differences are given as ITV − CMM; positive values indicate higher values in the ITV workflow. Target dose parameters are expressed as percentage of the prescription dose. For each parameter, the reported p-value is from the two-sided Wilcoxon signed-rank test; the adjusted p-value was additionally calculated using the Benjamini–Hochberg procedure, applied separately within four groups of comparisons: target volume dose (n = 22, 8 tests), plan quality (n = 22, 5 tests), three-fraction OAR parameters (n = 7, 24 tests), and five-fraction OAR parameters (n = 15, 24 tests). Adjusted p-values < 0.05 were considered statistically significant. Effect sizes are given as Hodges–Lehmann (HL) estimate of the paired difference with 95% confidence interval (not adjusted for multiple testing) and as rank-biserial correlation (r_rb_; ±1, all pairs differ in the same direction).

| **Parameter** | | **n** | **Median difference ITV − CMM [range]** | **HL estimate [95% confidence Interval]** | **r_rb_** | **p-value** | | **adjusted p-Value** | |
| --- | --- | --- | --- | --- | --- | --- | --- | --- | --- |
|  |  | **All plans** | | | | |  | |  |
|  |  | *Target volume and dose* | | | | |  | |  |
| PTV volume (cc) | | 22 | 10.11 [1.31, 40.68] | 13.32 [7.88, 20.15] | 1.00 | < 0.001 | | <0.001 | |
| GTV Dmean (%) | | 22 | −0.19 [−15.97, 4.08] | -0.67 [-2.57, 0.33] | -0.35 | 0.164 | | 0.219 | |
| GTV D98% (%) | | 22 | −0.08 [−18.71, 7.95] | -0.54 [-2.34, 0.96] | -0.19 | 0.443 | | 0.443 | |
| PTV D100% (%) | | 22 | −2.05 [−45.37, 5.70] | -2.08 [-7.04, -0.50] | -0.61 | 0.010 | | 0.020 | |
| PTV D98% (%) | | 22 | −0.13 [−25.54, 1.88] | -0.271 [-1.03, 0.28] | -0.21 | 0.406 | | 0.443 | |
| PTV D95% (%) | | 22 | −0.33 [−19.49, 2.10] | -0.44 [-1.58, 0.06] | -0.43 | 0.082 | | 0.131 | |
| PTV D50% (%) | | 22 | −1.75 [−11.20, 2.08] | -1.73 [-2.63, -0.81] | -0.77 | < 0.001 | | < 0.001 | |
| PTV D2% (%) | | 22 | −0.67 [−10.63, 4.44] | -0.95 [-2.24, -0.23] | -0.61 | 0.010 | | 0.020 | |
|  |  | **Plan quality** | | | | |  | |  |
| CI | | 22 | −0.03 [−0.58, 0.11] | −0.03 [−0.06, −0.01] | −0.50 | 0.039 | | 0.070 | |
| R50% | | 22 | −0.31 [−1.93, 0.84] | −0.30 [−0.61, −0.01] | −0.49 | 0.042 | | 0.070 | |
| GI | | 22 | −0.16 [−2.07, 6.12] | −0.10 [−0.38, 0.25] | −0.14 | 0.588 | | 0.588 | |
| V50% (cc) | | 22 | 54.0 [5.4, 269.2] | 68.2 [40.6, 106.9] | 1.00 | < 0.001 | | < 0.001 | |
| MU/Gy | | 22 | −0.05 [−13.96, 26.91] | 0.51 [−0.87, 3.38] | 0.17 | 0.503 | | 0.588 | |
|  |  | **Three-fraction plans** | | | | |  | |  |
| Liver-GTV Dmean (Gy) | | 7 | 0.97 [0.25, 2.12] | 0.97 [0.35, 1.70] | 1.00 | 0.016 | | 0.037 | |
| Liver D≥700cc (Gy) | | 7 | 0.54 [0.03, 0.94] | 0.36 [0.08, 0.77] | 1.00 | 0.016 | | 0.037 | |
| Liver-GTV V15Gy (cc) | | 7 | 31.50 [7.89, 115.0] | 31.81 [11.06, 77.74] | 1.00 | 0.016 | | 0.037 | |
| Liver-GTV V10Gy (%) | | 7 | 3.32 [0.44, 7.34] | 3.43 [0.73, 7.18] | 1.00 | 0.016 | | 0.037 | |
| Kidney R Dmean (Gy) | | 7 | 0.08 [0.00, 1.41] | 0.11 [0.01, 0.77] | 1.00 | 0.031 | | 0.058 | |
| Kidney R V6Gy (%) | | 7 | 0.00 [−2.95, 8.07] | 0 [-1.48, 4.04] | 0.33 | 0.750 | | 0.783 | |
| Chest wall D0.035cc (Gy) | | 7 | −0.17 [−2.24, 3.44] | 0.67 [-1.40, 2.74] | 0.21 | 0.688 | | 0.750 | |
| Chest wall D0.1cc (Gy) | | 7 | 1.85 [−1.85, 3.43] | 0.96 [-0.94, 2.74] | 0.50 | 0.297 | | 0.395 | |
| Chest wall D30cc (Gy) | | 7 | 1.08 [0.45, 3.25] | 1.09 [0.63, 2.22] | 1.00 | 0.016 | | 0.037 | |
| Bowel D0.1cc (Gy) | | 7 | 0.13 [−0.34, 4.58] | 0.33 [-0.16, 2.49] | 0.50 | 0.297 | | 0.395 | |
| Bowel D0.5cc (Gy) | | 7 | 0.59 [−0.11, 5.24] | 0.70 [0.01, 2.99] | 0.86 | 0.047 | | 0.075 | |
| Bowel D5cc (Gy) | | 7 | 0.77 [0.00, 5.03] | 0.84 [0.07, 2.95] | 1.00 | 0.031 | | 0.058 | |
| Stomach D0.1cc (Gy) | | 7 | 0.18 [−0.23, 1.03] | 0.18 [-0.11, 0.64] | 0.57 | 0.219 | | 0.328 | |
| Stomach D5cc (Gy) | | 7 | 0.41 [0.05, 6.16] | 0.41 [0.06, 3.31] | 1.00 | 0.016 | | 0.037 | |
| Stomach D10cc (Gy) | | 7 | 0.52 [0.05, 5.00] | 0.54 [0.16, 2.80] | 1.00 | 0.016 | | 0.037 | |
| Stomach D50cc (Gy) | | 7 | 0.48 [0.03, 1.47] | 0.46 [0.04, 1.08] | 1.00 | 0.016 | | 0.037 | |
| Duodenum D0.1cc (Gy) | | 7 | 0.24 [0.00, 4.65] | 0.42 [0.06, 2.57] | 1.00 | 0.031 | | 0.058 | |
| Duodenum D5cc (Gy) | | 7 | 0.09 [−1.00, 1.14] | 0.11 [-0.48, 0.67] | 0.52 | 0.312 | | 0.395 | |
| Duodenum D10cc (Gy) | | 7 | 0.03 [−0.73, 0.50] | 0.05 [-0.37, 0.30] | 0.33 | 0.625 | | 0.714 | |
| Esophagus D0.1cc (Gy) | | 7 | 0.01 [−0.89, 0.23] | 0.01 [-0.45, 0.14] | 0.07 | 0.938 | | 0.938 | |
| Esophagus D5cc (Gy) | | 7 | 0.02 [−0.24, 1.07] | 0.03 [-0.12, 0.56] | 0.36 | 0.469 | | 0.562 | |
| Heart D0.035cc (Gy) | | 7 | 0.10 [0.02, 0.59] | 0.10 [0.03, 0.44] | 1.00 | 0.016 | | 0.037 | |
| Heart D0.1cc (Gy) | | 7 | 0.07 [0.01, 0.65] | 0.09 [0.03, 0.58] | 1.00 | 0.016 | | 0.037 | |
| Heart D15cc (Gy) | | 7 | 0.03 [−0.02, 0.33] | 0.07 [0.01, 0.21] | 0.86 | 0.047 | | 0.075 | |
|  |  | **Five-fraction plans** | | | | |  | |  |
| Liver-GTV Dmean (Gy) | | 15 | 0.93 [−0.94, 3.75] | 1.08 [0.60, 1.59] | 0.87 | 0.002 | | 0.009 | |
| Liver D≥700cc (Gy) | | 15 | 0.55 [0.05, 2.84] | 0.70 [0.42, 1.21] | 1.00 | < 0.001 | | <0.001 | |
| Liver-GTV V15Gy (cc) | | 15 | 26.94 [7.90, 149.9] | 33.72 [20.80, 68.59] | 1.00 | < 0.001 | | <0.001 | |
| Liver-GTV V10Gy (%) | | 15 | 3.98 [−3.82, 9.22] | 3.82 [1.69, 5.49] | 0.88 | 0.001 | | 0.004 | |
| Kidney R Dmean (Gy) | | 15 | 0.14 [0.02, 8.75] | 0.28 [0.10, 2.01] | 1.00 | < 0.001 | | <0.001 | |
| Kidney R V6Gy (%) | | 15 | 1.00 [0.00, 22.06] | 0.92 [0.12, 4.91] | 1.00 | 0.008 | | 0.018 | |
| Chest wall D0.035cc (Gy) | | 15 | 0.05 [−1.72, 3.57] | 0.63 [-0.05, 1.61] | 0.47 | 0.124 | | 0.149 | |
| Chest wall D0.1cc (Gy) | | 15 | 0.06 [−1.79, 3.46] | 0.73 [-0.06, 1.58] | 0.47 | 0.124 | | 0.149 | |
| Chest wall D30cc (Gy) | | 15 | 1.28 [0.01, 9.97] | 1.32 [0.70, 2.47] | 1.00 | < 0.001 | | 0.003 | |
| Bowel D0.1cc (Gy) | | 15 | 0.36 [−1.21, 14.92] | 0.59 [-0.08, 1.64] | 0.55 | 0.064 | | 0.096 | |
| Bowel D0.5cc (Gy) | | 15 | 0.50 [−1.35, 13.96] | 0.67 [0.08, 4.17] | 0.62 | 0.035 | | 0.065 | |
| Bowel D5cc (Gy) | | 15 | 0.30 [−1.78, 12.86] | 0.79 [-0.31, 5.21] | 0.48 | 0.107 | | 0.143 | |
| Stomach D0.1cc (Gy) | | 15 | 0.12 [−2.39, 2.95] | 0.18 [-0.68, 0.93] | 0.25 | 0.421 | | 0.440 | |
| Stomach D5cc (Gy) | | 15 | 0.08 [−1.05, 2.02] | 0.09 [-0.41, 0.76] | 0.12 | 0.720 | | 0.720 | |
| Stomach D10cc (Gy) | | 15 | 0.17 [−1.06, 1.78] | 0.35 [-0.08, 0.89] | 0.43 | 0.151 | | 0.165 | |
| Stomach D50cc (Gy) | | 15 | 0.49 [−0.66, 1.39] | 0.45 [0.21, 0.72] | 0.83 | 0.006 | | 0.017 | |
| Duodenum D0.1cc (Gy) | | 15 | 0.200 [-1.490, 12.33] | 0.37 [0.04, 2.50] | 0.60 | 0.041 | | 0.071 | |
| Duodenum D5cc (Gy) | | 15 | 0.23 [0.01, 12.17] | 0.79 [0.15, 3.75] | 1.00 | < 0.001 | | 0.003 | |
| Duodenum D10cc (Gy) | | 15 | 0.21 [0.01, 3.54] | 0.39 [0.13, 1.24] | 1.00 | < 0.001 | | <0.001 | |
| Esophagus D0.1cc (Gy) | | 15 | 0.10 [−0.94, 9.02] | 0.50 [-0.10, 4.36] | 0.45 | 0.135 | | 0.155 | |
| Esophagus D5cc (Gy) | | 15 | 0.08 [−0.52, 5.98] | 0.413 [0, 1.79] | 0.60 | 0.055 | | 0.087 | |
| Heart D0.035cc (Gy) | | 15 | 0.08 [−0.22, 5.76] | 0.38 [0.03, 0.96] | 0.67 | 0.022 | | 0.046 | |
| Heart D0.1cc (Gy) | | 15 | 0.07 [−0.41, 6.23] | 0.20 [-0.04, 0.91] | 0.48 | 0.107 | | 0.143 | |
| Heart D15cc (Gy) | | 15 | 0.07 [−0.23, 3.55] | 0.12 [0.01, 0.78] | 0.71 | 0.023 | | 0.046 | |

n, number of evaluated plans; CI, conformity index (V_Rx / PTV volume); R50%, V_50% / PTV volume; GI, gradient index (V_50% / V_Rx); V_Rx and V_50%, body volumes receiving at least 100% and 50% of the prescription dose; MU, monitor units; Liver-GTV, uninvolved liver; R, right.

Supplementary Figure S1. Absolute parameter values of ITV-based and CMM plans for all parameters shown in Figure 1. Lines connect paired values from the same patient. p_adj_ shows the Benjamini–Hochberg adjusted p-value.
